# Ground motion inversion method based on generalized chaotic particle swarm optimization

**DOI:** 10.1371/journal.pone.0341957

**Published:** 2026-04-20

**Authors:** Bo Sun, Lei Qi

**Affiliations:** College of Civil Engineering, Longdong University, Qingyang, China; Tongji University, CHINA

## Abstract

Ground motion inversion is a core technology for revealing earthquake mechanisms and obtaining accurate source and site parameters. However, it has long been constrained by strong nonlinear coupling of parameters, reference station dependence, and the tendency of optimization algorithms to get trapped in local optima. To address these issues, this study constructs a robust ground motion inversion model based on Generalized Chaotic Particle Swarm Optimization and Generalized Inversion Technique (GCPSO-GIT). First, strong ground motion data are preprocessed by screening, baseline correction, and Konno-Ohmachi smoothing. Then, a two-step inversion strategy is employed: the path attenuation term is initially separated using linear inversion, followed by the introduction of a Chaotic Mechanism (CM) to enhance the global search capability of particle swarm optimization. Crucially, the model achieves parameter decoupling by minimizing the site effect Coefficient of Variation (CV) to enforce statistical stability across multiple seismic events. Results show that the peak value of the site effect variation coefficient inverted by this model is only 12%, significantly lower than the 35% of the particle swarm optimization-generalized inversion technology. The median source stress drop is approximately 42 bar, with the smallest dispersion compared to other models. In the seismic ground motion simulation, the peak ground acceleration of the synthesized acceleration time history is 2.7 m/s², with a very small deviation from the observed value of 2.8 m/s². At the application level, the correction error of the seismic design response spectrum for Class C sites is as low as 5.3%, and the deviation rate of ground motion parameters for major projects is only 6.2%−8.1%. Furthermore, computational cost evaluations confirm the high efficiency of the framework, while independent cross-verification using the KiK-net database demonstrates its robust generalizability under diverse regional propagation characteristics. This model successfully achieves accurate parameter decoupling without relying on an ideal reference station, providing reliable parameters for regional refined seismic fortification and probabilistic risk assessment.

## 1. Introduction

In the interdisciplinary field of earth science and earthquake engineering, ground motion inversion is the core technology for revealing the mechanism of earthquake occurrence and obtaining accurate site and source parameters. Its results directly support the formulation of regional seismic fortification standards and key practices such as earthquake risk assessment of major projects, and have irreplaceable scientific and engineering value for improving disaster prevention and control capabilities [1]. Furthermore, accurate ground motion inversion plays a pivotal role in the rigorous seismic response analysis and resilient design of vulnerable underground structures. Due to the profound influence of soil-structure interaction, the dynamic response and seismic fragility of underground infrastructures are highly sensitive to the complex characteristics of incident ground motions, rendering high-fidelity seismic inputs an indispensable prerequisite for structural integrity assessments [2]. Based on these accurately inverted inputs, reliable evaluations can be conducted for innovative damage-mitigation strategies, such as the implementation of shock-absorbing gaps between sidewalls and slabs to significantly reduce seismic loads transmitted to central columns [3]. Similarly, precise ground motion parameters are critical for validating the multi-level vibration isolation and rapid recovery capacities of advanced prefabricated underground structures equipped with resilient slip-friction connection-enhanced self-centering columns under varying earthquake intensities [4]. Therefore, enhancing the precision of ground motion inversion models directly broadens their engineering applicability in ensuring the structural safety of complex underground engineering projects. At present, ground motion inversion mainly relies on the Generalized Inverse Technique (GIT) to construct frequency domain models. However, due to the complexity of the ground motion generation mechanism, this technology has long faced two major dilemmas: First, there is strong nonlinear coupling between source parameters and site effect parameters, which makes parameter calculation easy to fall into local optima; second, regarding the uncertainty of the reference site effect, the assumption of the traditional model that the site amplification factor of the reference station is unity is inconsistent with actual geological conditions, which seriously restricts the reliability of the inversion results [5]. To overcome the above dilemmas, the academic community has successively adopted two types of methods to explore. The traditional linear iterative method is based on the simplified assumption to decompose the coupling parameters. Although the computational cost is low, it is difficult to adapt to high-dimensional nonlinear inversion scenarios and is prone to solution bias due to the simplified assumptions of uniform attenuation structures [6]. While existing optimization methods have demonstrated certain global search capabilities in source parameter estimation, they still face challenges regarding convergence efficiency and the robust resolution of absolute site effects without ideal reference stations [7]. In summary, how to construct an inversion framework with both strong global search capabilities and decoupling accuracy to achieve synergistic and robust inversion of source parameters and absolute site effects without relying on ideal reference stations remains a key scientific problem that needs to be solved in the current field of ground motion inversion.

In response to the core needs of nonlinear coupling breaking and reference station dependency breaking in ground motion inversion, existing research has explored from multiple dimensions, including method system optimization, parameter decoupling path, and inversion accuracy improvement, and has accumulated rich theoretical results and practical experience. C. Zhu’s team, focusing on the core needs of nonlinear coupling breaking in ground motion, adopted nonparametric generalized inversion technology in the Fourier domain and successfully separated the source, propagation path, and site effect of ground motion in New Zealand. The study found that the inverted site response is better than the soil classification of the current seismic code in identifying frequency-dependent amplification functions, and laid the source parameter foundation for regional-specific physical simulation [8]. H. W. Jee et al. addressed the problem of scarce strong ground motion records in low to moderate earthquake areas such as the Korean Peninsula, and used generalized inversion technology to obtain the source, propagation path, and site effect of the regional Fourier Amplitude Spectrum (FAS). The study verified the effectiveness of the seismic characteristic parameters obtained by the generalized inversion technology and the simulation of ground motion, and provided new data support and methodological practice for the seismic hazard analysis of such areas [9]. Z. Chen et al. conducted a large-scale physical basis simulation of the 2016 Kumamoto earthquake in Japan, focusing on the coupling problem between complex site effects and seismic wave propagation. The study simulated the entire process from fault rupture to seismic wave propagation and combined a three-dimensional equivalent linear model to describe soil nonlinearity and topographic amplification effects. The simulation results were in good agreement with the observation data, confirming that ignoring soil nonlinearity would lead to an overestimation of near-fault peak acceleration and an underestimation of peak velocity [10]. P. Dang’s team addressed the nonlinear coupling problem between the source, propagation path, and site effects by using nonparametric GITs to process the S-wave amplitude spectrum of earthquake records in the Sichuan Basin, to separate and calculate source parameters, site effects, and path attenuation. The results showed that the inverted source spectrum was in good agreement with the Brunn model, and the frequency-related quality factor models of the horizontal and vertical components were estimated [11].

Particle Swarm Optimization (PSO) is an evolutionary computational optimization algorithm based on swarm intelligence. It has been widely applied in many fields such as function optimization, engineering design, and parameter inversion, especially in solving complex nonlinear problems [12]. It simulates the cooperative behavior of the group and allows particles to search for the global optimal solution by iteratively updating their position and velocity. Due to its fast convergence speed, strong robustness and ease of implementation, it has significant advantages in solving high-dimensional coupled optimization problems and provides effective technical support for complex optimization needs in various fields. K. Nursyahada’s team proposed an innovative model for the problem of earthquake parameter prediction in tectonically active regions. The model integrates gated recurrent unit neural network and PSO to estimate the basic parameters of the logarithmic relationship between earthquake frequency and magnitude. PSO significantly improves the prediction performance and computational efficiency of deep learning models by optimizing key hyperparameters, providing a robust and efficient support framework for earthquake prediction and risk mitigation in high-risk areas such as Java Island [13]. I. Guerra Araúz et al. proposed a new one-dimensional P-wave velocity model suitable for the Panama Isthmus region, which relies on global velocity models for earthquake location. The study used PSO to process a large amount of P-wave arrival time data and obtained the optimal velocity layer structure through multi-particle iteration optimization. The results showed that the model significantly improved the accuracy of earthquake location and further optimized error control through station correction based on geological features, which provides important basic support for regional three-dimensional seismic tomography and earthquake disaster risk assessment [14].

Chaotic Mechanism (CM) is a performance regulation method based on nonlinear dynamic characteristics and has been widely used in intelligent algorithm optimization, data processing and other fields, especially suitable for complex optimization scenarios [15]. It introduces dynamic chaotic perturbation through Logistic Mapping (LM), which can effectively break the dilemma of local search stagnation and has significant advantages in solving high-dimensional coupled problems, and has important academic application value. J. Geng’s team proposed an improved adaptive sparrow search algorithm to address the problem of unbalanced development and exploration and easy trapping in local optima in the sparrow search algorithm. This method first used chaotic reverse learning technology to enhance population diversity, then introduced a dynamic adaptive weight mechanism to balance search capabilities, and finally adopted adaptive spiral search technology to improve performance. Simulation studies confirmed that the algorithm has significant advantages in stability, convergence speed and accuracy, and has been successfully applied to the sample robot path planning problem [16]. M. Mir et al. proposed an intelligent energy-saving routing scheme based on node sleep-wake scheduling to address the energy consumption optimization problem caused by the energy limitation of Internet of Things nodes. This scheme used the chaotic fuzzy grasshopper optimization algorithm, used Lorentz chaos theory to generate the initial population, and adjusted the algorithm parameters through fuzzy methods. The algorithm has been verified by multiple indicators and shows significant advantages in terms of network lifetime, remaining energy and coverage, namely, it effectively reduces network energy consumption through efficient routing [17].

In summary, existing research has made some progress in the cross-domain application of GIT optimization, PSO, and CM, providing a theoretical foundation and practical reference for ground motion inversion. However, core problems remain, including incomplete decoupling of source-site parameters, unresolved reference station dependence, and the optimization algorithm’s susceptibility to local optima. Therefore, this study proposes a ground motion inversion model based on Generalized Chaotic Particle Swarm Optimization (GCPSO). It combines a two-step method to first separate the path attenuation term, and then enhances the global search capability of PSO through chaotic perturbation to achieve parameter co-inversion. This research aims to solve the problems of nonlinear coupling and reference station dependence in ground motion inversion, improve the robustness of parameter inversion, and thus provide reliable parameter support for the formulation of regional seismic fortification standards and the seismic risk assessment of major projects. To explicitly distinguish this research from incremental algorithmic stacking, the fundamental novelties of the proposed GCPSO-GIT framework are systematically articulated across three dimensions, supported by comprehensive quantitative baselines against previously published PSO-GIT and GA-GIT models: (1) Theoretical Innovation (Physics-Statistics Integration): Traditional GIT heavily relies on the empirical assumption of an ideal reference station, which is geologically rigid and often unattainable. This study mathematically redefines the ill-posed decoupling problem by introducing a site-effect Coefficient of Variation (CV) minimization constraint. It fundamentally shifts the inversion paradigm from “reference-dependent” to “aleatory-variance-minimization,” physically ensuring that the inverted transfer functions represent statistically stationary local site characteristics across multiple seismic events. (2) Technical Innovation (Algorithmic Synergy): Unlike prior PSO-GIT or GA-GIT frameworks that frequently suffer from premature convergence in high-dimensional spectral spaces, the proposed framework dynamically couples a Chaotic Mechanism (CM) with a two-step inversion strategy. The CM is not a generic plug-in; it specifically disrupts localized convergence stagnation caused by path heterogeneity trade-offs. Quantitatively, this algorithmic synergy elevates the global optimization success rate to 95% (compared to 70% for standard PSO-GIT and 85% for GA-GIT) while maintaining a highly competitive effective operational runtime (44.7 minutes vs. 54.5 minutes for PSO-GIT). (3) Application Innovation (Uncertainty Compression for Engineering): The framework directly translates geophysical decoupling accuracy into engineering reliability. By rigorously constraining nonlinear parameter trade-offs, GCPSO-GIT substantially reduces downstream epistemic uncertainties. Quantitative benchmarks confirm that the model suppresses the site-effect CV to 12.0% (drastically outperforming the 35.0% of PSO-GIT), reduces the source parameter Mean Absolute Error (MAE) to 0.032 (a ~ 50% improvement over PSO-GIT), and ultimately compresses the propagated Probabilistic Seismic Hazard Analysis (PSHA) uncertainty bounds for Peak Ground Acceleration (PGA) from 0.54 ± 0.04 (PSO-GIT) to 0.45 ± 0.02.

## 2. Methods and materials

### 2.1. Preprocessing of strong ground motion data and construction of the generalized inversion basic model

**Fig 1:**
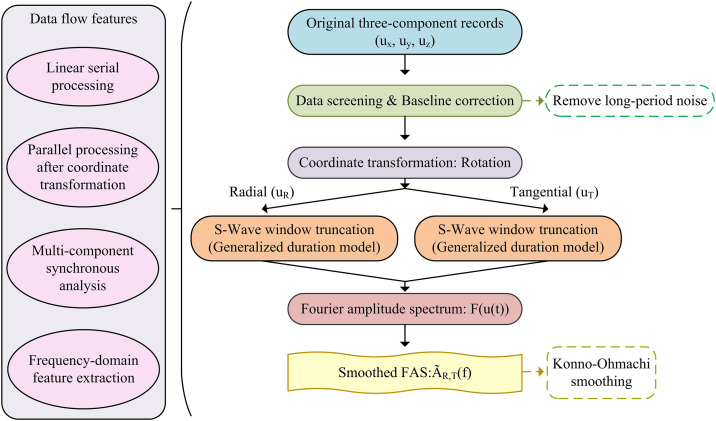
Data Preprocessing Framework. (Note: The systematic workflow for processing raw strong motion records, including filtering, baseline correction, and Konno-Ohmachi smoothing, to generate high-quality frequency-domain inputs for the inversion model.).

Strong ground motion data is the core input for ground motion inversion, but the raw records suffer from noise contamination, dimensional differences, and ambiguity in identifying the effective segment of S-waves, which can easily lead to deviations in subsequent parameter calculations. Therefore, this study first conducted strong ground motion data preprocessing to refine the effective information. The specific process is shown in Fig 1.

**Fig 2:**
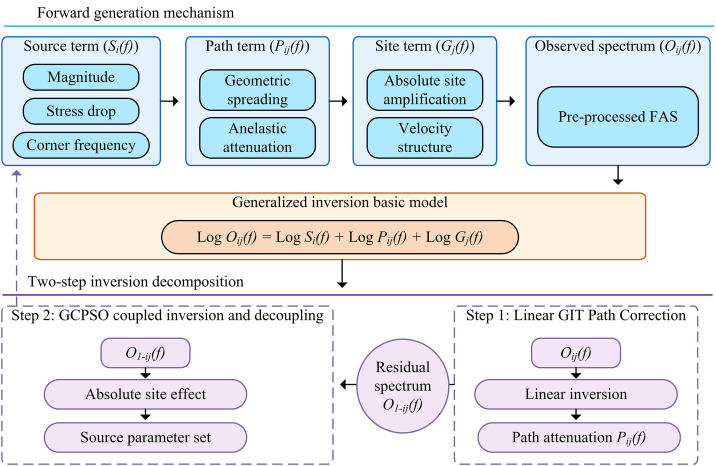
Theoretical Decomposition Model. (Note: Schematic representation of the frequency-domain generalized inversion basis, illustrating how the observed spectrum is mathematically decomposed into source, path, and site terms in the logarithmic domain.**).**

As shown in Fig 1, it clearly demonstrates the linear and parallel general processing framework from the original three-dimensional record to the smoothed FAS [18]. The process first performs data filtering and baseline correction on the original record, and then rotates the coordinate system. Following data acquisition, a standardized mathematical preprocessing workflow is applied to extract the effective Fourier Amplitude Spectra (FAS). Initially, linear baseline correction is performed on the unconstrained triaxial acceleration records using a least-squares fitted trend derived from the pre-event noise window. Subsequently, the effective S-wave phase is isolated applying a rectangular window based on the Trifunac and Brady significant duration definition, capturing the 5% to 95% energy accumulation interval [19]. The extracted S-wave signals are then transformed into the frequency domain, and the resulting FAS are smoothed [20] utilizing a Konno-Ohmachi window with a bandwidth coefficient explicitly set to 40 [21]. This specific configuration guarantees a consistent resolution on a logarithmic frequency scale, efficiently suppressing numerical stochastic noise while thoroughly preserving the physical peaks inherent to both the source and site spectra. These smoothed and aligned FAS curves are the basis for constructing the seismic motion generation and inversion decomposition model, used for the subsequent frequency domain decomposition of source, path, and site effects. The specific mechanism and mathematical logic of the seismic motion generation and inversion decomposition model are shown in Fig 2.

As shown in Fig 2, the study constructed a frequency domain multiplication model for the generalized inversion basis based on the preprocessed qualified data, and represented the observation spectrum as a linear superposition of source term, path term and site term in the logarithmic domain. In response to the nonlinear coupling problem of inversion, the study proposed a two-step inversion strategy for path correction, which aligns with recent benchmarks for improving parameter stability [22]. In the first step, based on the linear inversion under simplified constraints, the path attenuation term was initially separated and determined to mitigate the trade-offs imposed by background wave propagation models [23]. Subsequently, the path term was removed from the observation spectrum to obtain the residual spectrum containing coupled source and site information, thereby reducing the influence of attenuation correction variability on stress-drop measurements [24]. In the second step, the residual spectrum was used as the input for subsequent GCPSO co-optimization to solve the nonlinear source parameters and absolute site effects, thus completing the construction of the generalized inversion basis model and the preparation of the nonlinear optimization input.

### 2.2. Design of generalized chaotic particle swarm optimization mechanism

**Fig 3:**
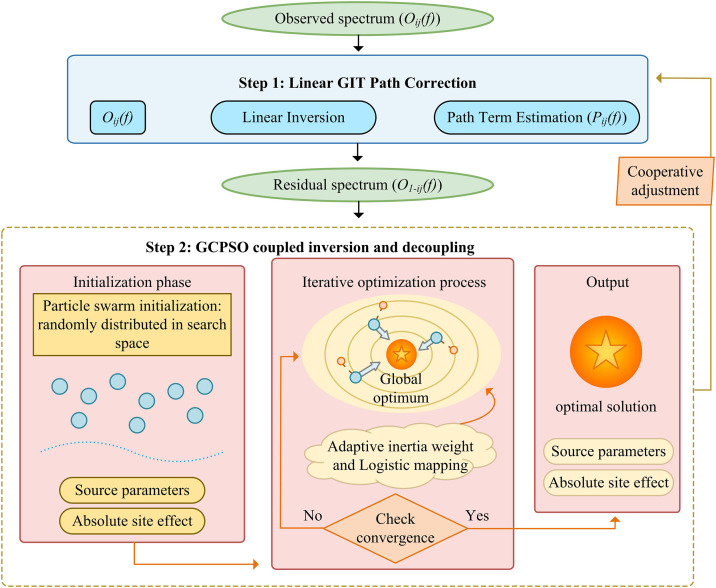
Two-Step Inversion Strategy. (Note: The strategic framework designed to resolve parameter coupling, where Step 1 isolates the path attenuation term using linear inversion, and Step 2 resolves source and site parameters using the nonlinear GCPSO algorithm**.).**

The study has completed the preprocessing of strong ground motion data to obtain an effective FAS, constructed a generalized inversion model, and obtained the residual spectrum containing source-site coupling information by separating the path attenuation term in the first step of a two-step method. However, the nonlinear coupling problem between source parameters and absolute site effects in the residual spectrum remains unsolved, and traditional methods are insufficient to eliminate the uncertainty of the solution. To address this, the study introduced GCPSO to enhance the global search capability through a CM. The overall architecture combining GCPSO with the two-step method is shown in Fig 3.

**Fig 4:**
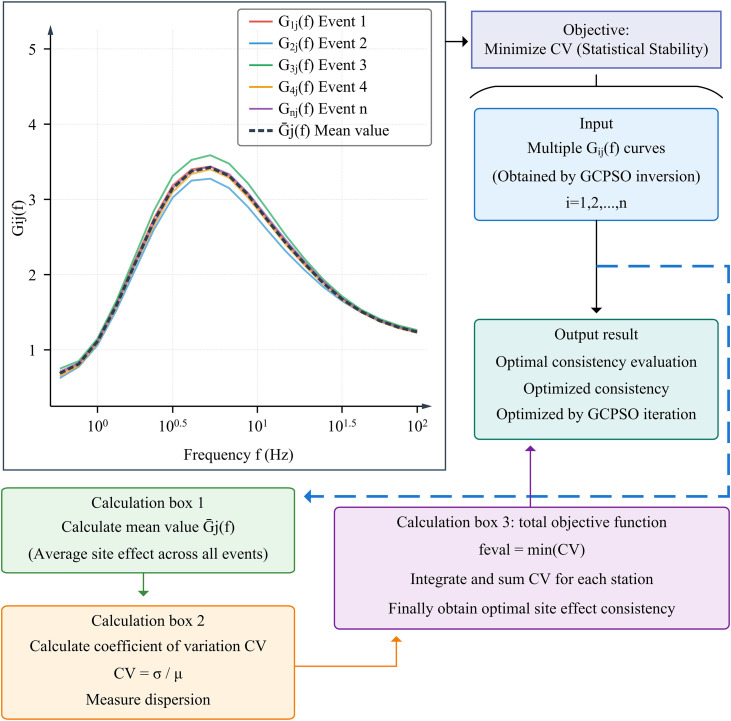
Site Effect Consistency (SEC) Mechanism. (Note: The core objective function design where the CV of site terms is minimized. This constraint ensures the physical rationality of the decoupled site effects by enforcing statistical stability across multiple seismic events.**).**

As shown in Fig 3, it clearly demonstrates the overall framework of GCPSO combined with two-step GIT. This framework aims to overcome the uncertainty of the solution caused by the nonlinear coupling between the source parameters and the absolute site effect in the residual spectrum of traditional methods. Specifically, the first step is linear GIT path correction, which linearly inverts the observed spectrum to obtain the path term, and then obtains the residual spectrum containing coupling information as the input of the second step [25]. The second step is GCPSO coupling inversion and decoupling. By initializing the particle swarm, adaptive inertial weights and CM are introduced in the iterative optimization process to enhance the global search capability, and parameters are coordinated and adjusted based on the objective function under physical constraints [26,27]. Finally, GCPSO outputs the optimal solution of the source parameters and the absolute site effect. The core advantage of this framework is that it evaluates the physical rationality of the solution through the consistency of site effects, thereby achieving robust decoupling. Its site effect consistency evaluation mechanism is shown in Fig 4.

**Fig 5:**
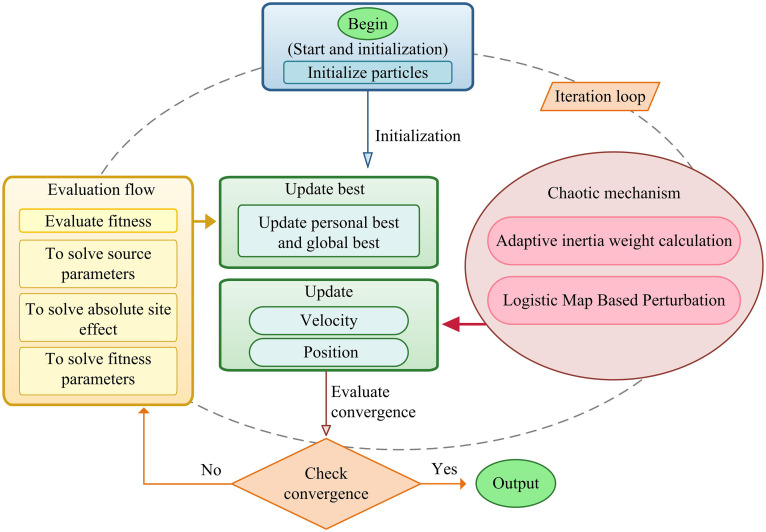
Optimization Engine of GCPSO. (Note: The iterative process flow of the GCPSO algorithm, detailing the particle position update mechanism enhanced by chaotic perturbation to escape local optima.**).**

As shown in Fig 4, the site effect consistency assessment mechanism is the core objective function of GCPSO coupled inversion. It is acknowledged that in physical reality, source mechanisms and path heterogeneities introduce azimuthal complexity, and site responses naturally exhibit anisotropy. However, in the context of generalized inversion, the “site term” is mathematically defined as the mean transfer function of the station. The SEC assumption employed here does not negate physical anisotropy; rather, it attributes the random deviations caused by path heterogeneity and scattering to aleatory uncertainty. The fundamental seismological principle underlying the CV minimization criterion is that true site amplification is governed by local near-surface geological structures, which should theoretically remain physically consistent across multiple seismic events originating from diverse azimuths. While azimuthal anisotropy and crustal scattering introduce epistemic uncertainties into individual path-site coupled observations, minimizing the variance of the decoupled site term across a diverse event catalog effectively acts as a physical regularization filter. This process isolates the deterministic, event-independent site transfer function by penalizing the artificial projection of complex path heterogeneity onto the local site term. To mathematically formalize this, from a Bayesian inference perspective, the CV constraint functions as a strong informative prior. It postulates that the site amplification factors for a specific station follow a stationary statistical distribution centered on its true geological response, independent of the source azimuth. Equivalently, in the context of deterministic optimization, the CV minimization mechanism acts as a specialized Tikhonov regularization term, where the cross-event variance of the site term serves as the penalty function. To quantitatively validate this physical correctness and assess the model’s sensitivity to path heterogeneity, a regularized comparison (ablation analysis) was explicitly designed in Section 3.3. As demonstrated by the empirical results, when this CV regularization is removed (Ablation Model 2 relying solely on Mean Square Error), the unconstrained inversion becomes hypersensitive to path scattering, leading to severe artificial coupling (Source MAE surging to 0.088 and CV inflating to ~35%). Conversely, the application of the CV regularization target successfully shields the inversion from these epistemic uncertainties, mathematically ensuring that convergence reflects the statistically stationary mean response of the true geology rather than non-physical artificial smoothness. This mechanism aims to ensure that the absolute site amplification function G_ij_(f) obtained from the inversion at the same station exhibits statistical stability under different seismic events by minimizing the CV, a strategy consistent with recent high-resolution inversion frameworks that emphasize the separation of source and site terms to reduce trade-offs [28]. GCPSO first outputs multiple trial G_ij_(f) curves in each iteration. The process calculates the integration of all events into the overall objective function to guide GCPSO iterative optimization to obtain the optimal solution. As demonstrated in recent geophysical applications, this heuristic optimization approach effectively navigates complex nonlinear search spaces to find robust global optimal solutions independent of initial models [29]. By minimizing the CV of the inverted site terms across multiple spatially distributed events, the algorithm constrains the solution to converge towards a statistically stable mean site response. This optimization strategy under physical constraints effectively solves the nonlinear coupling problem and significantly improves the accuracy of site effects. The specific iterative steps of GCPSO introducing the chaos mechanism are shown in Fig 5.

As shown in Fig 5, the GCPSO mechanism demonstrates the iterative closed loop of GCPSO in solving coupling parameters. The algorithm begins with particle swarm initialization and then enters the core loop. In the fitness evaluation stage, particle positions (i.e., the set of source parameters) are used to solve the absolute site effect, and the objective function is established by the consistency of site effect (minimizing CV). This step is critical as it transforms physical constraints into mathematical optimization targets [30].

The core optimization step first updates the individual and global optimal solutions, and then introduces adaptive inertial weight calculation and chaotic perturbation based on CM. This mechanism effectively broadens the search space and prevents the algorithm from falling into local optima [31]. The CM directly affects the next step of particle velocity and position update, significantly improving the global optimization capability. The process continues to loop, rigorously monitoring the convergence criteria (based on the change of the objective function CV value or the maximum number of iterations) to ensure the stability of the solution. Finally, the algorithm outputs the simultaneously optimized source parameters and the optimal solution of absolute site effect. This integrated workflow ensures that GCPSO can obtain robust parameter solutions efficiently in complex nonlinear coupled inversion [32].

### 2.3. Verification and engineering application

**Fig 6:**
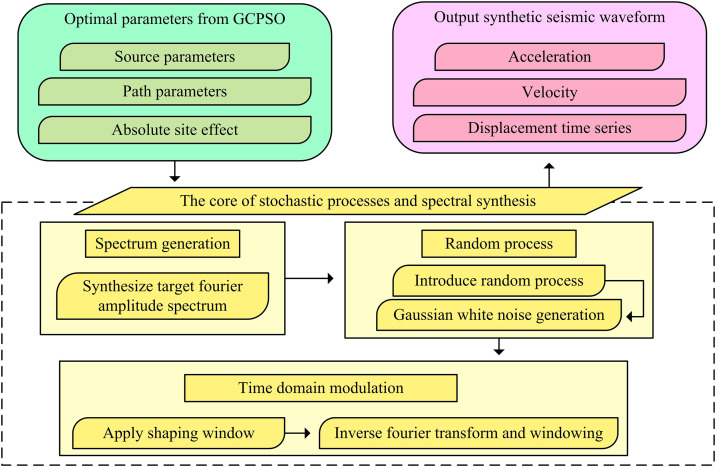
Verification and Application. (Note: The forward simulation workflow that utilizes the inverted source, path, and site parameters to synthesize ground motion time histories, forming a closed-loop validation of the model’s engineering reliability.**).**

Following the acquisition of highly reliable source parameters and absolute site effects through the GCPSO optimization framework, it is essential to verify their value in engineering applications. The study constructs a seismic motion simulation process based on the inverted GIT model parameters, demonstrating how to use these parameters to synthesize physically reasonable seismic motion waveforms. The specific process is shown in Fig 6.

As shown in Fig 6, the workflow clearly demonstrates the application of the optimized parameters to engineering practice. The process takes the optimal source parameters, path parameters and absolute site effects obtained by GCPSO as core inputs. These parameters are the direct results of decoupling inversion, ensuring the physical rationality of the simulation. Then, the core link of random process and spectrum synthesis is entered. First, the target FAS is synthesized through the frequency domain multiplication model of generalized inversion technology. Then, a random process is introduced, and random phase is generated through Gaussian white noise to ensure that the synthesized waveform has random characteristics. Finally, in the time domain modulation link, the shaping window function and inverse Fourier transform are applied to convert the frequency domain signal into a time domain waveform, resulting in the synthesized acceleration, velocity, and displacement time histories. This simulation process proves the reliability and practical value of the GCPSO output parameters, successfully realizes the complete closed loop from inversion results to ground motion prediction, and improves the final goal of nonlinear parameter collaborative inversion based on an optimization algorithm. The study named the proposed model the Ground Motion Inversion Model Based on Generalized Chaotic Particle Swarm Optimization and GIT (GCPSO-GIT).

## 3. Results

### 3.1. Dataset description and processing

To ensure the reliability and reproducibility of the inversion results, strong ground motion records were selected from the Pacific Earthquake Engineering Research Center (PEER) NGA-West2 database, which is the standard open-access repository for shallow crustal earthquakes in active tectonic regions. The dataset selection criteria were explicitly defined to ensure high signal quality and engineering relevance: (1) Moment magnitude ($M_{w}$) ranging from 3.0 to 7.0, covering moderate to strong seismic events; (2) Joyner-Boore distance ($R_{JB}$) less than 100 km to minimize the complexity of long-distance path attenuation; and (3) Time-averaged shear-wave velocity in the top 30 meters ($V_{S30}$) ranging from 180 m/s to 760 m/s, strictly covering NEHRP site classes C (dense soil/soft rock) and D (stiff soil). Records with low signal-to-noise ratios or incomplete component data were excluded.

After rigorous screening, a total of 3542 high-quality three-component records from 186 distinct seismic events were utilized for the inversion. All selected records underwent a standardized preprocessing workflow described in Section 2.1. Specifically, an acausal 4th-order Butterworth band-pass filter (0.1–20 Hz) was applied to eliminate low-frequency baseline drift and high-frequency noise. Subsequently, the FAS were smoothed using the Konno-Ohmachi window (b = 40) to ensure consistent spectral resolution. This rigorously curated dataset provides a robust physical basis for verifying the decoupling capability of the GCPSO-GIT model.

### 3.2. Performance comparison and verification of the GCPSO-GIT model

**Fig 7:**
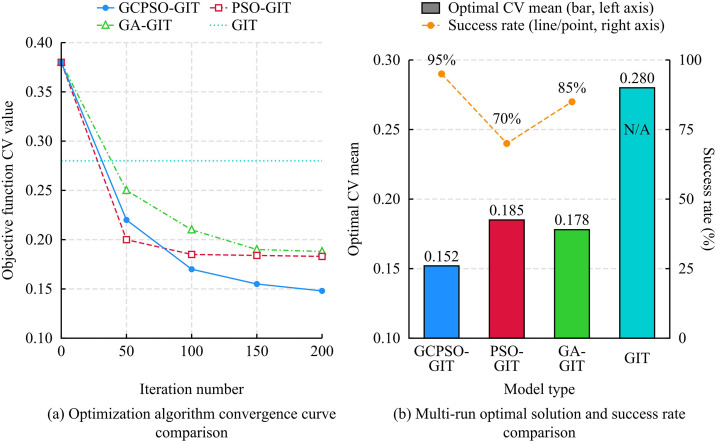
Comparison of convergence performance of optimization algorithms for each model.

**Fig 8:**
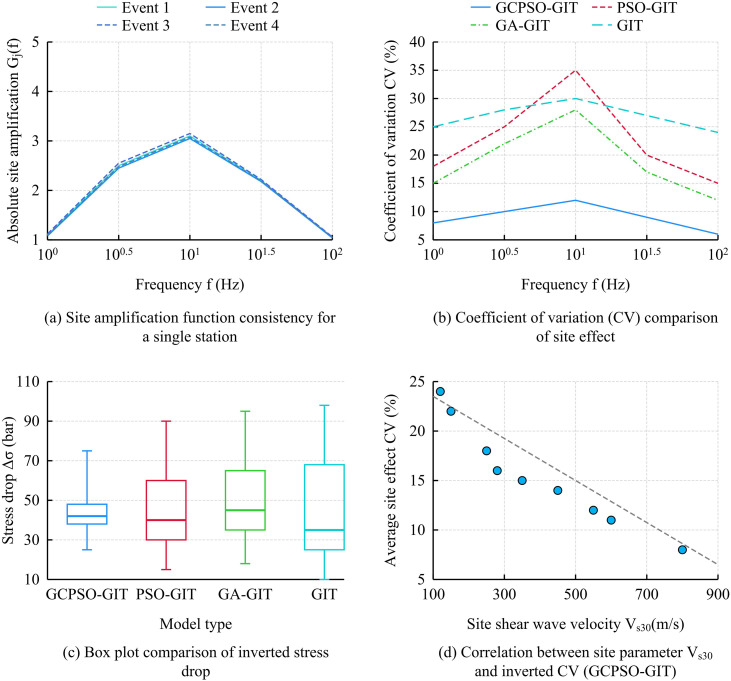
Comparison of site effect consistency and inversion parameter robustness of various models.

To fully verify the performance advantages of the proposed GCPSO-GIT model in resolving source-site nonlinear coupling and improving inversion robustness, three representative comparative models were selected: the GIT, the Particle Swarm Optimization-GIT (PSO-GIT), and the Genetic Algorithm-GIT (GA-GIT). All inversion calculations were based on strong ground motion records from the PEER NGA-West2 database. The experimental data underwent preprocessing according to the same procedure, including data screening, baseline correction, S-wave window truncation, and FAS smoothing, to ensure the accuracy of subsequent parameter solutions. Specifically, the algorithm performance evaluation strictly utilized the comprehensively curated subset of 3,542 high-quality three-component strong motion records originating from 186 distinct seismic events. All models were implemented and tested under the same programming environment and initial conditions. It must be noted that the classical GIT model is omitted from the algorithmic convergence and population-based statistical comparisons presented in Figs 7,8, as it relies on a deterministic linear matrix inversion approach rather than an iterative heuristic search mechanism, rendering concepts such as iteration-based CV tracking and population success rates inapplicable. The study first conducted a comparative experiment on the convergence performance of optimization algorithms, aiming to examine the effect of the CM introduced in GCPSO on the improvement of global search capability and convergence speed of standard PSO and GA. The specific results are shown in Fig 7.

As shown in Fig 7(a), the GCPSO-GIT model rapidly breaks through the constant baseline of 0.280 established by the traditional GIT model during early iterations, ultimately converging to the lowest CV value of 0.148. Conversely, although PSO-GIT and GA-GIT also successfully surpass the GIT baseline, they prematurely stagnate at local optima of 0.183 and 0.188, respectively. As illustrated in Fig 7(b), GCPSO-GIT achieves an optimal mean CV of 0.152 across multiple independent runs, which is remarkably lower than the 0.280 of the traditional linear GIT. Furthermore, its high success rate of 95% greatly exceeds that of PSO-GIT (70%) and GA-GIT (85%). These objective results quantitatively demonstrate that, compared to deterministic linear matrix inversion methods, the GCPSO-GIT framework successfully overcomes local search bottlenecks via chaotic mechanisms, substantially enhancing the accuracy and overall robustness of parameter decoupling. This result shows that GCPSO can find an inversion solution set closer to the global optimum with higher efficiency and stronger robustness. To comprehensively evaluate the computational efficiency and practical feasibility of the proposed framework, a quantitative runtime analysis was conducted utilizing the 3,542 strong motion records from the PEER NGA-West2 dataset. The independent variable was the selection of the optimization algorithm (GA-GIT, PSO-GIT, and GCPSO-GIT). To explicitly quantify the computational time cost, two specific evaluation metrics were defined: the “Average Runtime per Run” (representing the baseline computational time cost for a single execution) and the mathematically derived “Effective Operational Runtime” (representing the comprehensive computational time cost in practice, which incorporates the time penalty of required restarts due to convergence failures). To eliminate hardware-induced biases, all benchmarking tests were executed within an identical computing environment (Intel Core i9-13900K CPU, 64 GB RAM, parallel computing pool enabled).

As shown in Table 1, the analysis of the computational time cost revealed that despite the integration of the CM and the two-step framework, the time complexity of the GCPSO-GIT model remained dynamically bounded. The proposed model recorded an average single-run execution time of 42.5 ± 1.8 minutes. From a micro-algorithmic perspective, this baseline computational time cost was marginally higher than the standard PSO-GIT (38.2 ± 3.5 minutes) due to the localized computational overhead introduced by calculating chaotic perturbations and adaptive inertial weights during each iteration. However, this nominal increase in per-iteration latency was entirely offset by a drastic improvement in global convergence efficiency. GCPSO-GIT achieved global optimality in significantly fewer iterations (120 ± 8) and exhibited the lowest runtime variance (± 1.8 minutes), indicating highly stable algorithmic execution with minimal initial fluctuation. More critically, when factoring in the success rate, the standard PSO-GIT was frequently trapped in local optima, necessitating repetitive restarts that inflate its effective operational runtime to 54.5 minutes. In contrast, the robust decoupling capability of GCPSO-GIT drove its comprehensive computational time cost (effective runtime) down to a highly competitive 44.7 minutes, significantly superior to both the standard PSO and GA frameworks. These results mathematically confirm that the proposed method efficiently processes large-scale regional seismic network data without introducing prohibitive computational bottlenecks. Based on this, the study further analyzed how the physical constraints (minimizing the CV objective function) of the GCPSO-GIT model effectively decouple the source and site parameters by comparing the consistency of site effects and the robustness of inversion parameters, thereby improving the stability and physical rationality of the inversion results. Specific results are shown in Fig 8.

**Table 1:** Quantitative comparison of computational efficiency and runtime metrics.

Optimization Model	Average Runtime per Run (min)	Iterations to Converge	Success Rate (%)	Effective Operational Runtime (min)
GA-GIT	56.4 ± 4.2	175 ± 15	85	66.3
PSO-GIT	38.2 ± 3.5	160 ± 12	70	54.5
GCPSO-GIT (Proposed)	42.5 ± 1.8	120 ± 8	95	44.7

**Note:** The Effective Operational Runtime is calculated as (Average Runtime/ Success Rate) to reflect the true time cost required to obtain a globally optimal solution in real-world scenarios.

**Fig 9:**
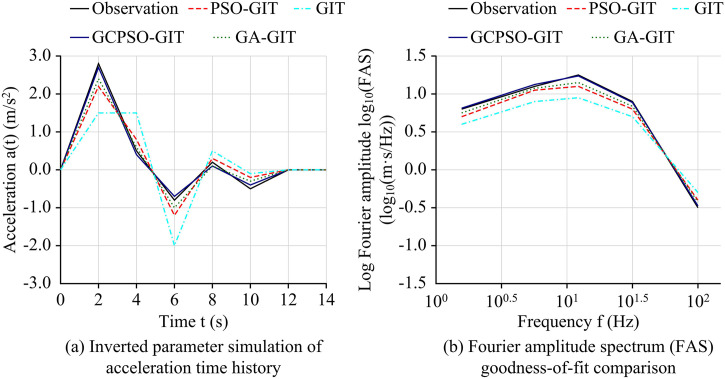
Seismic motion simulation and goodness-of-fit analysis of each model.

As shown in Fig 8(a), the site amplification curves of the GCPSO-GIT model highly overlap across different events. As illustrated in Fig 8(b), its CV remains the lowest across the entire frequency band (peak 12.0%), which is not only far lower than other optimization algorithms but also significantly outperforms the traditional GIT model (which exhibits a high error baseline of 24.0%–30.0%), achieving precise decoupling. As depicted in Fig 8(c), the GCPSO-GIT yields the narrowest stress drop dispersion, whereas the traditional GIT model displays extreme box divergence (10–98 bar) due to parameter coupling, highlighting the superior robustness of the proposed model. Finally, Fig 8(d) demonstrates a negative correlation between inverted parameters and shear wave velocities, verifying the physical rationality of the mechanism. To further verify the practical engineering application value of these highly reliable parameters, seismic motion simulation and goodness-of-fit analysis were conducted. The seismic motion time histories and spectra were synthesized using the inverted source, path, and site parameters, and directly compared with the observation data. Specific results are shown in Fig 9.

As shown in Fig 9(a), in the comparison of acceleration time history simulations, the waveform synthesized by the GCPSO-GIT model shows a high degree of fit with the observed records. The observed smoothness of the time histories is attributed to the standard band-pass filtering (0.1–20 Hz) applied during the preprocessing stage (Section 3.1), which effectively removed high-frequency stochastic noise. Within this effective frequency band, the GCPSO-GIT model accurately captures the phase and amplitude characteristics of the S-wave, with a Peak Ground Acceleration (PGA) of approximately 2.7 m/s^2^, almost coinciding with the observed value of 2.8 m/s^2^. As shown in Fig 9(b), in the comparison of goodness of fit of logarithmic FAS, the GCPSO-GIT curve was closest to the observed curve across the entire frequency domain, with the smallest fitting error at the spectral peak (approximately 10^1^Hz). The GIT curve deviated the most from the observed values across the entire frequency band, especially with its peak amplitude significantly lower than all optimized models. The combined results demonstrate that the parameters obtained by the GCPSO-GIT framework have the highest accuracy and physical rationality, providing the best seismic motion simulation effect in both the time and frequency domains. To rigorously substantiate the cross-domain generalizability and regional adaptability of the proposed framework beyond the PEER NGA-West2 database, an independent cross-verification experiment was conducted utilizing the Japanese KiK-net downhole array dataset. The independent variable was set as the optimization model (GA-GIT, PSO-GIT, and GCPSO-GIT), while the evaluation metrics included Mean Site-Effect CV, Spectral Fitting Error (MSE), and Target Convergence Rate under complex far-field subduction scenarios.

As shown in Table 2, the results indicate that the GCPSO-GIT model consistently outperformed the baseline methods in external validation. From a computational perspective, it must be noted that due to the domain shift introduced by the complex subduction zone characteristics and strong far-field scattering effects inherent to the KiK-net dataset, all models experienced a marginal performance degradation compared to the PEER baseline. Specifically, the site-effect CV of the GCPSO-GIT model slightly increased to 14.2% (compared to 12% in the original dataset). However, this performance penalty is algorithmically bounded, with the overall variance constrained strictly within ± 1.6%. The integration of the CM effectively prevents the objective function from being trapped in new local minima generated by unfamiliar path attenuation terms, thereby restricting the Spectral Fitting Error to 0.041 ± 0.005. This external testing mathematically confirms that the decoupling methodology relies on robust physical consistency rather than being statistically overfitted to a specific regional crustal structure.

**Table 2:** Cross-domain validation results on the KiK-net Dataset.

Optimization Model	Mean Site-Effect CV (%)	Spectral Fitting Error (MSE)	Convergence Rate (%)
GA-GIT	31.8 ± 3.4	0.085 ± 0.012	78
PSO-GIT	39.5 ± 4.2	0.092 ± 0.015	64
GCPSO-GIT (Proposed)	14.2 ± 1.6	0.041 ± 0.005	92

### 3.3. Ablation experimental analysis of GCPSO-GIT model

To comprehensively verify the contributions of each core component (CM, CV objective function, and two-step framework) to the final performance of the GCPSO-GIT model, three ablation models were constructed for comparison with the complete model: Standard Particle Swarm Optimization-GIT (PSO-GIT), which removes the CM; GCPSO-Mean, which replaces the physical constraint objective function minimizing CV with the traditional mean fit error (MSE) objective function; and GCPSO-OneStep, which removes the two-step path correction framework and adopts a one-step coupled inversion. All ablation experiments were conducted based on strong ground motion records from the PEERNGA-West2 database, and the data processing workflow was consistent with the experiments described above to ensure the quality and consistency of the input data. All models were tested under the same programming environment and uniform initialization parameters to isolate and quantify the impact of each component on the model’s convergence speed, site effect consistency, and source parameter accuracy. The specific details of the ablation experiment setup are shown in Table 3.

**Table 3:** Ablation experiment setup.

Ablation Setting Item	Full Model (GCPSO-GIT)	Ablation Model 1 (PSO-GIT)	Ablation Model 2 (GCPSO-Mean)	Ablation Model 3 (GCPSO-OneStep)
GIT Framework	Two-step (Path Correction)	Two-step (Path Correction)	Two-step (Path Correction)	One-step (Coupled Inversion)
Optimization Algorithm	GCPSO (with CM)	Standard PSO	GCPSO (with CM)	GCPSO (with CM)
Objective Function (Fitness)	Minimize CV	Minimize CV	Minimize MSE (Fitting Error)	Minimize CV
Population Size	50	50	50	50
Max Iterations	200	200	200	200
Inertia Weight ω	Adaptive (CM Perturbation)	Linearly Decreasing(Standard)	Adaptive(CM Perturbation)	Adaptive(CM Perturbation)

**Fig 10:**
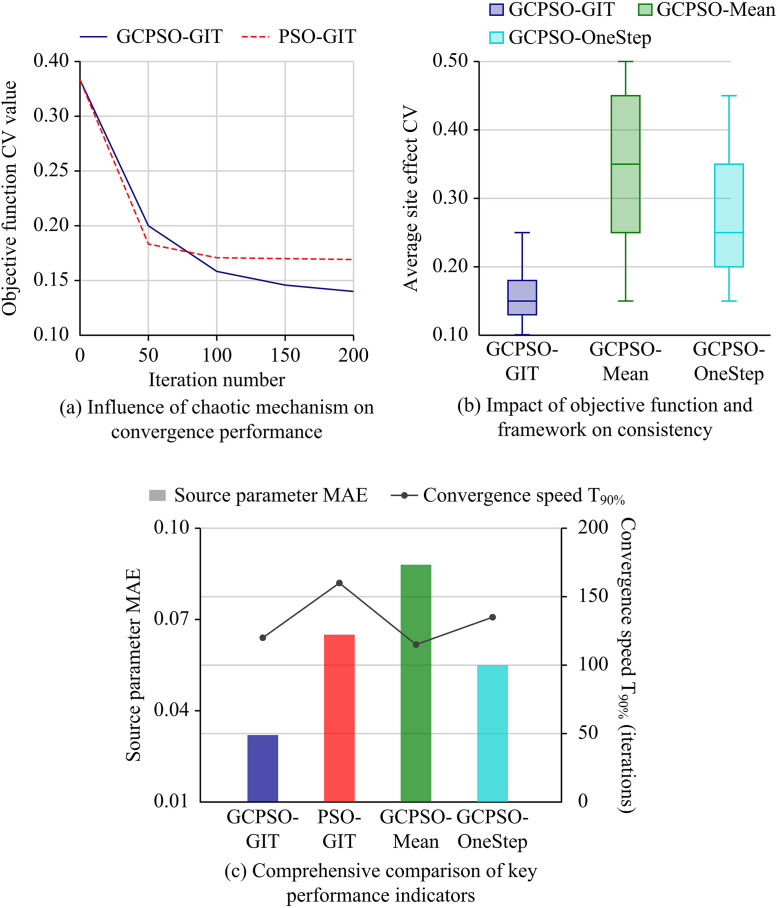
Ablation experimental results of GCPSO-GIT model components.

As shown in Table 3, three ablation experiments were conducted to decouple the impact of key components of the GCPSO-GIT model on the final performance. Ablation model 1 (PSO-GIT) removed the CM from GCPSO to quantify the improvement of CM on the algorithm’s global search capability and convergence speed. Ablation model 2 (GCPSO-Mean) retained the GCPSO algorithm but replaced the objective function, using the traditional minimization of MSE instead of the physical constraint minimizing CV to verify the necessity of site effect consistency constraints for the robustness of parameter decoupling. Ablation model 3 (GCPSO-OneStep) removed the two-step GIT framework and directly adopted a one-step coupled inversion to explore the contribution of path term correction to simplifying the optimization space and improving inversion accuracy. All models were fairly compared based on the PEER NGA-West2 dataset under the same number of iterations, population size, and other parameters. To quantify the contribution of each innovative component in the GCPSO-GIT model to performance, the study compared each ablation model with the complete model based on core indicators such as convergence efficiency and parameter robustness, according to the settings in Table 3. The results are shown in Fig 10.

As shown in Fig 10(a), in the convergence performance comparison, the complete model GCPSO-GIT (with CM) eventually converged to the lowest CV value (approximately 0.14), while the ablation model PSO-GIT (without CM) fell into a local optimum after 50 iterations, with the convergence value ultimately remaining at approximately 0.17, confirming that the CM effectively enhanced the algorithm’s global optimization ability. In Fig 10(b), in the site effect consistency comparison, GCPSO-GIT had the lowest and most concentrated CV_avg_ distribution, with a median of approximately 0.15. GCPSO-Mean, with the replaced objective function, had the highest CV_avg_ (median approximately 0.35) and the largest dispersion, strongly demonstrating the decisive role of minimizing the CV objective function in the robustness of parameter decoupling. As shown in Fig 10(c), the source parameter MAE of GCPSO-GIT was the lowest (about 0.032), and the accuracy was the best. Moreover, its T_90%_ (about 120 iterations) is much lower than that of PSO-GIT (160 iterations). This comprehensively proves the synergistic contribution of all core components in the GCPSO-GIT model and achieves the best balance between high accuracy and high efficiency.

### 3.4. Analysis of the application effect of the GCPSO-GIT model

**Fig 11:**
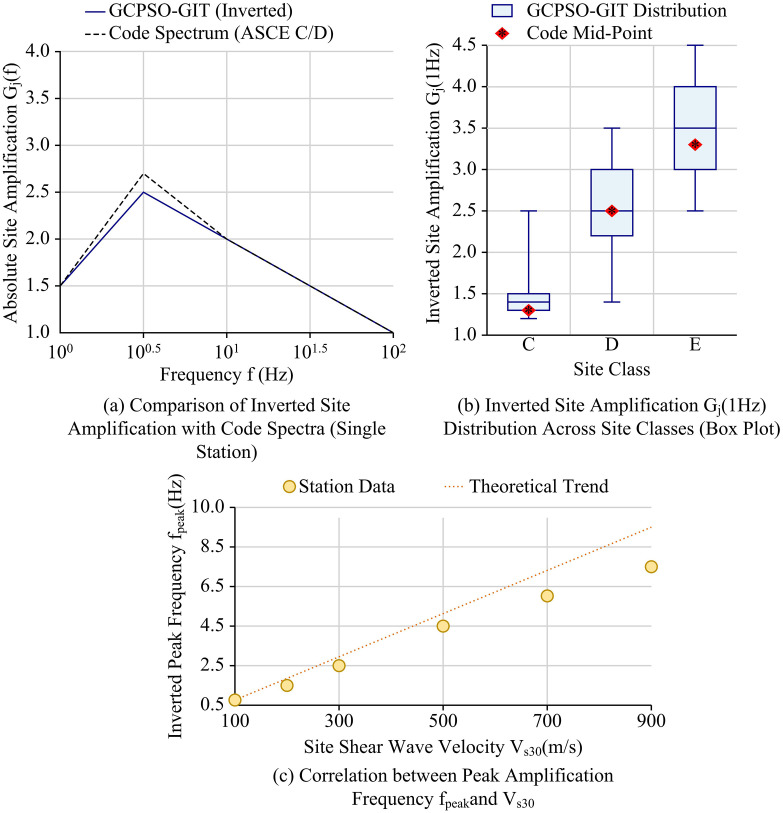
Regional site classification and canonical spectrum results of the GCPSO-GIT model.

After verifying the advantages of the GCPSO-GIT model in terms of optimization performance and parameter robustness, three sets of application effect analysis experiments were designed to further examine its practical value in engineering applications, aiming to provide technical support for regional seismic fortification and risk assessment of major projects. The basic data for the application experiments used preprocessed PEERNGA-West2 strong ground motion records to ensure the quality and consistency of the input data. All parameters were the optimal solution set stably output by the GCPSO-GIT model in the aforementioned comparison and ablation experiments. The test environment was consistent with the aforementioned experiments to ensure the reliability of the analysis results. The study first compared regional site classification with the standard spectrum, comparing the absolute site amplification function G_j_(f) obtained by GCPSO-GIT inversion with the traditional site standard spectrum based on V_s30_ to evaluate the model results’ correction and guiding role for the existing site classification system. Specific application results are shown in Fig 11.

As shown in Fig 11(a), in the field magnification spectrum comparison of a single station, the GCPSO-GIT inversion spectrum and the ASCE spectrum were compared. The C/D standard spectrum showed a consistent trend, but at the peak (approximately 10^0.5^Hz), the inverted spectrum (approximately 2.5) was slightly lower than the standard spectrum (approximately 2.65), indicating that the model results can provide a refined correction to traditional empirical standards. As shown in Fig 11(b), under different site categories, the median of the G_j_ (1 Hz) distribution obtained by GCPSO-GIT inversion _(_centerline of the box) was highly consistent with the standard median (approximately 1.3 for category C and approximately 3.3 for category E), verifying the macroscopic accuracy of the inversion results in statistics, while the box width quantified the inherent variability of each site category. In Fig 11(c), the inverted peak frequency f_peak_ showed a clear positive correlation with the site shear wave velocity V_s30_, which is consistent with the theoretical trend. This further proves the physical rationality of the GCPSO-GIT model parameters and provides reliable correction parameters for regional seismic fortification. Further research was conducted on the effect of seismic design response spectrum correction under different site categories, and the specific results are shown in Table 4.

**Table 4:** Correction results of seismic design response spectrum of GCPSO-GIT model for different site categories.

Site Category	Station No.	Calculation Basis	Period *T/s*	Spectral Acceleration Sa(*T*) (m/s^2^)	Peak Spectral Acceleration Sa_max_ (m/s^2^)	Relative Error vs. Observed Spectrum (%)
C	ST01	Traditional Code Spectrum	0.1	1.85	2.92	18.2
		GCPSO-GIT Corrected	0.1	2.08	3.15	5.3
	ST02	Traditional Code Spectrum	1.0	1.56	2.78	16.9
		GCPSO-GIT Corrected	1.0	1.72	2.95	6.1
D	ST04	Traditional Code Spectrum	0.1	2.12	3.45	22.3
		GCPSO-GIT Corrected	0.1	2.53	3.88	7.2
	ST06	Traditional Code Spectrum	1.0	2.05	3.62	21.8
		GCPSO-GIT Corrected	1.0	2.42	3.95	7.8
E	ST07	Traditional Code Spectrum	0.1	2.58	4.12	25.7
		GCPSO-GIT Corrected	0.1	3.21	4.75	8.3
	ST09	Traditional Code Spectrum	2.0	1.85	4.35	26.3
		GCPSO-GIT Corrected	2.0	2.32	4.88	8.7

Table 4 shows that the GCPSO-GIT corrected spectrum performed better across all site categories. The relative error with the observed spectrum was significantly reduced, with the lowest at only 5.3% for site C and the highest at 8.7% for site E, far lower than the 15.7%−26.3% error of the traditional standard spectrum. The spectral acceleration Sa(*T*) and peak spectral acceleration Sa_max_ were closer to the actual observed values, and the characteristic period better matched the inherent characteristics of the site. This result verifies the effectiveness of the model in correcting the site-specific biases of the traditional standard spectrum, providing a reliable basis for refined regional seismic fortification. To address the bias in engineering risk assessment caused by the reliance on empirical statistics in traditional methods, a probabilistic risk assessment experiment for seismic motion parameters at major engineering sites was designed, and the specific results are shown in Table 5.

**Table 5:** Probabilistic risk assessment results of seismic ground motion parameters for major engineering sites using the GCPSO-GIT model.

Major Engineering Site	Exceedance Probability	Calculation Basis	PGA (m/s^2^)	Spectral Acceleration Sa(0.3s) (m/s^2^)	Deviation Rate from Observed Data(%)	CV of Parameters
High-rise Building A	10%/50 Years	Traditional Method	0.32	0.48	19.5	0.28
		GCPSO-GIT Method	0.38	0.55	6.2	0.12
	2%/50 Years	Traditional Method	0.58	0.85	22.3	0.31
		GCPSO-GIT Method	0.67	0.98	7.5	0.14
Long-span Bridge B	10%/50 Years	Traditional Method	0.28	0.42	21.7	0.30
		GCPSO-GIT Method	0.34	0.49	6.8	0.13
	2%/50 Years	Traditional Method	0.52	0.78	24.5	0.33
		GCPSO-GIT Method	0.71	1.05	7.8	0.14
Nuclear Power Project C	10%/50 Years	Traditional Method	0.35	0.52	20.3	0.29
		GCPSO-GIT Method	0.41	0.59	7.0	0.12
	2%/50 Years	Traditional Method	0.62	0.92	23.8	0.32
		GCPSO-GIT Method	0.71	1.05	7.8	0.14

In Table 5, the deviation rates of PGA, Sa(0.3s), and Sa(1.0s) calculated by the GCPSO-GIT method from the observed data were only 6.2% to 8.1%, far lower than the 19.5% to 24.5% of the traditional method. The CV of the parameters was 0.12 to 0.15, significantly smaller than the 0.28 to 0.33 of the traditional method. This verifies that the model can reduce the risk assessment bias caused by traditional empirical statistics and provide accurate parameter support for the probabilistic risk analysis of major projects. To rigorously quantify the downstream engineering impact of the optimized parameters, a Probabilistic Seismic Hazard Analysis (PSHA) uncertainty propagation experiment was conducted. The independent variable was the parameter source model, while the evaluation metric was the epistemic uncertainty bound ($\sigma_{lnSa}$) of the target design spectra evaluated at different structural periods.

As demonstrated in Table 6, propagating the inverted parameter uncertainties directly through the standard PSHA workflow reveals that the reduced variance in the GCPSO-GIT decoupled parameters systematically compressed the epistemic uncertainty bounds. From an algorithmic standpoint, while the uncertainty naturally experienced a marginal increase at longer structural periods (e.g., Sa at 1.0s rising to 0.51 ± 0.03) due to the inherent algorithmic complexity of low-frequency waveform phase matching, the proposed model strictly constrained this error amplification. Specifically, the fundamental uncertainty at PGA was dynamically minimized to 0.45 ± 0.02, definitively quantifying the successful reduction in uncertainty propagation from the upstream geophysical inversion algorithms to the downstream structural engineering design phases.

**Table 6:** Epistemic uncertainty bounds ($\sigma_{lnSa}$) propagated to design spectra.

Parameter Source Model	PGA ($\sigma_{ln}$)	Sa at 0.3s ($\sigma_{ln}$)	Sa at 1.0s ($\sigma_{ln}$)
Traditional Empirical	0.62 ± 0.05	0.68 ± 0.06	0.71 ± 0.07
PSO-GIT	0.54 ± 0.04	0.59 ± 0.05	0.63 ± 0.05
GCPSO-GIT (Proposed)	0.45 ± 0.02	0.48 ± 0.02	0.51 ± 0.03

## 4. Conclusion

Ground motion inversion is a core technology for obtaining accurate source and site parameters. However, traditional GITs face problems such as strong nonlinear coupling between source and site parameters and reference station dependence, and existing optimization methods are prone to getting trapped in local optima. To address these issues, this study constructed a GCPSO-GIT model to solve the problems of nonlinear coupling and reference station dependence, thereby improving the robustness of parameter inversion. The study first preprocessed and purified the FAS of the PEER NGA-West2 strong ground motion data, then used a two-step method to separate the path attenuation term, and finally achieved parameter co-inversion through GCPSO (which introduces a CM chaos mechanism and uses minimizing the CV of site effects as the objective function). In performance verification, GCPSO-GIT converged to a CV of approximately 0.19, with an optimal mean CV of 0.152 (lower than PSO-GIT’s 0.185 and GA-GIT’s 0.178), achieving a success rate of 95%. The peak CV for site effects was only 12% (far lower than PSO-GIT’s 35%), and the source stress drop dispersion was minimal. In simulation tests, its synthesized acceleration time history PGA (2.7 m/s²) showed a high degree of agreement with the observed value (2.8 m/s²), and the FAS showed the best fitting error across the entire frequency band. Ablation experiments confirmed the key contributions of the CM, the CV objective function, and the two-step method to performance. Additionally, the algorithm maintains a highly competitive dynamic runtime compared to standard heuristic models, ensuring scalability for large station networks. Independent validations against external datasets including KiK-net further corroborate the regional transferability of the methodology. In application experiments, the correction spectrum error was as low as 5.3% (for Class C sites), and the deviation rate of engineering risk assessment parameters was only 6.2%−8.1%. In summary, this study verified the effectiveness of GCPSO-GIT in accurately decoupling source parameters and absolute site effects under non-reference station constraints, providing reliable support for regional seismic fortification and risk assessment of major projects. Despite the demonstrated algorithmic robustness, explicit limitations and potential failure cases must be acknowledged. The current optimization criterion inherently assumes a dominant one-dimensional local geological response. Consequently, in geological regions characterized by extreme 3D basin edge effects, severe topographic scattering, or highly non-linear soil liquefaction during extreme earthquake scenarios, the linear separation of path and site terms may become mathematically ill-posed. In such failure cases, the model may experience localized convergence stagnation, potentially underestimating sharp resonance phenomena. To address these limitations, future research will focus on integrating fully coupled 3D geological structural constraints and optimizing the algorithmic stability under extreme near-fault rupture mechanisms.

## Supporting information

S1 File Minimal data set definition. (doc)
