# Supplementary material for: Ground motion inversion method based on generalized chaotic particle swarm optimization
Source: PLoS One. 2026 Apr 20;21(4):e0341957. doi: 10.1371/journal.pone.0341957 (PMC13094990; doi:10.1371/journal.pone.0341957)
Supplement: S1 File — (doc) [file pone.0341957.s001.doc]

Minimal Data Set for Figure 7(a): Optimization algorithm convergence curve comparison

| Iteration Number | GCPSO-GIT (Objective Function CV Value) | PSO-GIT (Objective Function CV Value) | GA-GIT (Objective Function CV Value) |
| --- | --- | --- | --- |
| 0 | 0.380 | 0.380 | 0.380 |
| 50 | 0.220 | 0.200 | 0.250 |
| 100 | 0.170 | 0.185 | 0.210 |
| 150 | 0.155 | 0.184 | 0.190 |
| 200 | 0.148 | 0.183 | 0.188 |

Minimal Data Set for Figure 7(b): Multi-run optimal solution and success rate comparison

| Model Type | Optimal CV Mean | Success Rate (%) |
| --- | --- | --- |
| GCPSO-GIT | 0.152 | 95 |
| PSO-GIT | 0.185 | 70 |
| GA-GIT | 0.178 | 85 |

**Minimal Data Set for Figure 8(a): Site amplification function consistency for a single station (GCPSO-GIT)**

| Frequency f (Hz) | Log(f) | Event 1 G_j_(f) | Event 2 G_j_(f) | Event 3 G_j_(f) | Event 4 G_j_(f) |
| --- | --- | --- | --- | --- | --- |
| 1.00 | 0.0 | 1.10 | 1.08 | 1.12 | 1.09 |
| 3.16 | 0.5 | 2.50 | 2.45 | 2.55 | 2.48 |
| 10.00 | 1.0 | 3.10 | 3.05 | 3.15 | 3.08 |
| 31.60 | 1.5 | 2.20 | 2.18 | 2.22 | 2.19 |
| 100.00 | 2.0 | 1.05 | 1.04 | 1.06 | 1.05 |

Minimal Data Set for Figure 8(b): Coefficient of variation (CV) comparison of site effect

| Frequency f (Hz) | Log(f) | GCPSO-GIT CV (%) | PSO-GIT CV (%) | GA-GIT CV (%) |
| --- | --- | --- | --- | --- |
| 1.00 | 0.0 | 8.0 | 18.0 | 15.0 |
| 3.16 | 0.5 | 10.0 | 25.0 | 22.0 |
| 10.00 | 1.0 | 12.0 | 35.0 | 28.0 |
| 31.60 | 1.5 | 9.0 | 20.0 | 17.0 |
| 100.00 | 2.0 | 6.0 | 15.0 | 12.0 |

Minimal Data Set for Figure 8(c): Box plot comparison of inverted stress drop

| Model Type | Minimum (bar) | First Quartile Q1 (bar) | Median (bar) | Third Quartile Q3 (bar) | Maximum (bar) |
| --- | --- | --- | --- | --- | --- |
| GCPSO-GIT | 25 | 38 | 42 | 48 | 75 |
| PSO-GIT | 15 | 30 | 40 | 60 | 90 |
| GA-GIT | 18 | 35 | 45 | 65 | 95 |

Minimal Data Set for Figure 8(d): Correlation between site parameter Vs30 and inverted CV (GCPSO-GIT)

| Station ID | Site Shear Wave Velocity Vs30 (m/s) | Average Site Effect CV (%) |
| --- | --- | --- |
| Station 1 | 150 | 22.0 |
| Station 2 | 250 | 18.0 |
| Station 3 | 350 | 15.0 |
| Station 4 | 450 | 14.0 |
| Station 5 | 800 | 8.0 |
| Station 6 | 120 | 24.0 |
| Station 7 | 600 | 11.0 |
| Station 8 | 280 | 16.0 |
| Station 9 | 550 | 12.0 |
| Station 10 | 950 | 6.0 |

Minimal Data Set for Figure 9(a): Inverted parameter simulation of acceleration time history

| Time t (s) | Observation a(t) (m/s^2^) | GCPSO-GIT a(t) (m/s^2^) | PSO-GIT a(t) (m/s^2^) | GA-GIT a(t) (m/s^2^) | GIT a(t) (m/s^2^) |
| --- | --- | --- | --- | --- | --- |
| 0 | 0.00 | 0.00 | 0.00 | 0.00 | 0.00 |
| 2 | 2.80 | 2.70 | 2.20 | 2.40 | 1.50 |
| 4 | 0.50 | 0.40 | 0.80 | 0.60 | 1.50 |
| 6 | -0.80 | -0.70 | -1.20 | -1.00 | -2.00 |
| 8 | 0.20 | 0.10 | 0.30 | 0.10 | 0.50 |
| 10 | -0.50 | -0.40 | -0.20 | -0.30 | -0.10 |
| 12 | 0.00 | 0.00 | 0.00 | 0.00 | 0.00 |
| 14 | 0.00 | 0.00 | 0.00 | 0.00 | 0.00 |

Minimal Data Set for Figure 9(b): Fourier amplitude spectrum (FAS) goodness-of-fit comparison

| Data Point | Observation Log10(FAS) | GCPSO-GIT Log10(FAS) | PSO-GIT Log10(FAS) | GA-GIT Log10(FAS) | GIT Log10(FAS) |
| --- | --- | --- | --- | --- | --- |
| Point 1 | 0.80 | 0.81 | 0.70 | 0.75 | 0.60 |
| Point 2 | 1.10 | 1.13 | 1.05 | 1.08 | 0.90 |
| Point 3 | 1.25 | 1.24 | 1.10 | 1.15 | 0.95 |
| Point 4 | 0.90 | 0.89 | 0.80 | 0.84 | 0.70 |
| Point 5 | -0.50 | -0.48 | -0.40 | -0.43 | -0.30 |

Minimal Data Set for Figure 10(a): Influence of chaotic mechanism on convergence performance

| Iteration Number | GCPSO-GIT Objective Function CV Value | PSO-GIT Objective Function CV Value |
| --- | --- | --- |
| 0 | 0.380 | 0.380 |
| 50 | 0.220 | 0.200 |
| 100 | 0.170 | 0.185 |
| 150 | 0.155 | 0.184 |
| 200 | 0.148 | 0.183 |

Minimal Data Set for Figure 10(b): Impact of objective function and framework on consistency (Box Plot)

| Model Type | Minimum CV | First Quartile Q1 | Median CV | Third Quartile Q3 | Maximum CV |
| --- | --- | --- | --- | --- | --- |
| GCPSO-GIT | 0.10 | 0.13 | 0.15 | 0.18 | 0.25 |
| GCPSO-Mean | 0.15 | 0.25 | 0.35 | 0.45 | 0.50 |
| GCPSO-OneStep | 0.15 | 0.20 | 0.25 | 0.35 | 0.45 |

Minimal Data Set for Figure 10(c): Comprehensive comparison of key performance indicators

| Model Type | Source Parameter MAE | Convergence Speed T90% (iterations) |
| --- | --- | --- |
| GCPSO-GIT | 0.032 | 120 |
| PSO-GIT | 0.065 | 160 |
| GCPSO-Mean | 0.088 | 115 |
| GCPSO-OneStep | 0.055 | 135 |

Minimal Data Set for Figure 11(a): Comparison of inverted site amplification with code spectra (Single Station)

| Frequency f (Hz) | Log(f) | GCPSO-GIT (Inverted) Absolute Site Amplification G_j_(f) | Code Spectrum (ASCE C/D) Absolute Site Amplification G_j_(f) |
| --- | --- | --- | --- |
| 1.00 | 0.0 | 1.50 | 1.50 |
| 3.16 | 0.5 | 2.50 | 2.70 |
| 10.00 | 1.0 | 2.00 | 2.00 |
| 31.60 | 1.5 | 1.50 | 1.50 |
| 100.00 | 2.0 | 1.00 | 1.00 |

**Minimal Data Set for Figure 11(b): Inverted site amplification G_j_**(1Hz) distribution across site classes (Box Plot)

| Site Class | Minimum | First Quartile Q1 | Median | Third Quartile Q3 | Maximum | Code Mid-Point |
| --- | --- | --- | --- | --- | --- | --- |
| C | 1.20 | 1.30 | 1.40 | 1.50 | 2.50 | 1.30 |
| D | 1.40 | 2.20 | 2.50 | 3.00 | 3.50 | 2.50 |
| E | 2.50 | 3.00 | 3.50 | 4.00 | 4.50 | 3.30 |

**Minimal Data Set for Figure 11(c): Correlation between peak amplification frequency f_peak_ and V_s30_**

| Station Data Point | Site Shear Wave Velocity V_s30_ (m/s) | Inverted Peak Frequency f_peak_ (Hz) |
| --- | --- | --- |
| Station 1 | 100 | 0.76 |
| Station 2 | 200 | 1.50 |
| Station 3 | 300 | 2.50 |
| Station 4 | 500 | 4.50 |
| Station 5 | 700 | 6.03 |
| Station 6 | 900 | 7.50 |
